# Ingestion of High-Oleic Peanut Improves Endurance Performance in Healthy Individuals

**DOI:** 10.1155/2022/3757395

**Published:** 2022-03-10

**Authors:** Morimasa Kato, Mayuko Omiya, Makino Horiuchi, Daisuke Kurata

**Affiliations:** ^1^Department of Health and Nutrition, Yamagata Prefectural Yonezawa University of Nutrition Sciences, Yonezawa 992-0025, Japan; ^2^Bean Research Section, Denroku Co Ltd, 3-2-45 Kiyozumi-Machi, Yamagata 990-8506, Japan

## Abstract

This study aimed at evaluating whether high-oleic peanuts (with skin), which are rich in oleic acid, could serve as an energy substrate for prolonged exercise and improve endurance performance. We evaluated changes in blood biomarker (triglycerides, free fatty acid (FFA), biological antioxidant potential (BAP), malondialdehyde-modified low-density lipoprotein (MDA-LDL), and serum total protein) levels at 2-h intervals for 6 h after the ingestion of 10 g and 30 g of peanuts. The results were used to determine the timing of peanut ingestion before the endurance performance test. As a result, there was a significant change in the 30-g peanut-ingested condition, and lipid levels increased 2 h after the ingestion of 30 g of peanuts. Accordingly, the endurance performance test was conducted 2 h after ingesting 30 g of peanuts. The endurance performance test involved 70 min of pedaling exercise. We measured pre- and postexercise levels of 8-hydroxy-2′-deoxyguanosine (8-OHdG), which is a biomarker of oxidative stress. There was a significantly improved workload in the endurance performance test in the high-oleic peanut-ingested condition than in the control condition. Furthermore, the rate of increase in 8-OHdG was significantly lower in the high-oleic peanut-ingested condition than in the control condition. This suggests that the increase in FFA levels resulting from the ingestion of high-oleic peanuts and the inherent antioxidant effects of peanuts improved the workload during endurance exercise.

## 1. Introduction

In prolonged exercise, it is important to keep the steady supply of energy needed throughout the activity. This energy demand is met by the degradation of adenosine triphosphate, and the major energy supply systems for resynthesis are anaerobic and aerobic energy supply systems [1], with lipids as the primary energy substrate in the exercise duration [2].

Studies on improving endurance performance using supplements have mainly focused on glycogen storage [3], thermoregulation [4, 5], water retention [6], proton accumulation in active muscles [7], and exercise-induced oxidative stress [8]. There have also been studies on the acute effects of ingesting fatty acids (FAs), which are the primary energy substrates for endurance exercise; however, they have not demonstrated any improvement in exercise performance [9, 10]. Nonetheless, Esquius et al. recently reported that ingesting olive oil and almonds as sources of FAs improved cardiorespiratory coordination and endurance performance [11, 12]. Notably, almond ingestion was found to improve endurance performance. Furthermore, foods containing both FAs and polyphenols may contribute to endurance improvement.

Although peanuts are legumes, they have similar nutritional characteristics as nuts. Moreover, there has been increasing evidence regarding the potential health benefits of high-oleic peanut cultivars, which are richer in oleic acid than conventional peanut cultivars [13–16]. Oleic acid-rich peanuts increase insulin production and reduce diabetic symptoms in type II diabetic mice [16]. Studies on continuous ingestion by humans have shown no changes in body composition [13] and improved cognitive function [14]. High-oleic peanuts contain high levels of oleic acid, which is a FA that can be a useful energy source for lipid metabolism, as well as vitamin E and polyphenols [13], which could contribute to enhanced exercise performance.

We hypothesized that the ingestion of high-oleic peanuts (with skin) would serve as an energy substrate for lipid metabolism during exercise, reduce exercise-induced oxidative stress, and improve exercise performance. Accordingly, we aimed at investigating the effect of ingesting high-oleic peanuts on blood lipid kinetics and endurance performance.

## 2. Materials and Methods

### 2.1. Experiment 1

#### 2.1.1. Participants

We included eight healthy female participants (age: 21.6 ± 0.5 years, height: 160.3 ± 4.6 cm, weight: 56.3 ± 2.8 kg, body mass index [BMI]: 21.9 ± 1.0 kg/m^2^, complete blood count [CBC]: white blood cells [WBCs] 4738.0 ± 1082/*μ*L, red blood cells [RBCs] 435 ± 23.1 × 104 *μ*L, hemoglobin [Hgb] 12.3 ± 1.7 g/dL, hematocrit [Hct] 39.0 ± 3.7%, and platelets [PLT] 28.4 ± 10.5 × 104 *μ*L), who provided informed consent to participate in the experiment. All participants were normotensive without any neurological, cardiovascular, pulmonary, or kidney diseases. Their CBC values were in the normal range, and they did not take any medications or supplements daily. Additionally, all participants were students at dietitian training schools and had dietary lifestyles that reflect a comprehensive understanding of the dietary reference intakes for Japanese. This study was approved by the Ethics Committee of the Yamagata Prefectural Yonezawa University of Nutrition Sciences (No. 28-11). The trial registration number was UMIN000044446.

#### 2.1.2. Test Materials

This study used high-oleic peanuts cultivated in the USA. The obtained high-oleic peanuts were roasted (unsalted) at a confectionery company (Denroku Co Ltd. Japan), and the samples that were safe for human ingestion were used in the experiment. The high-oleic peanuts used in this study were assessed using gas chromatography and had a composition as follows: palmitic acid (16 : 0): 5.7%, stearic acid (C18 : 0): 1.5%, oleic acid (C18 : 1): 79.2%, linoleic acid (C18 : 2): 5.2%, alpha-linolenic acid (C18 : 3): 0.1%, arachidic acid (C20 : 0): 0.8%, eicosenoic acid (C20 : 1): 2.6%, behenic acid (C22 : 0): 2.7%, erucic acid (C22 : 1): 0.3%, and lignoceric acid (C24 : 0): 1.9%. Total polyphenols were assessed by the Folin–Ciocalteu assay and was 830 mg/100 g, and vitamin E was assessed by high-performance liquid chromatography and was 4.8 mg total, with 0.4 mg/100 g *α*-tocopherol, 0.4 mg/100 g *β*-tocopherol, 7.7 mg/100 g *γ*-tocopherol, and 0.9 mg/100 g *δ*-tocopherol.

#### 2.1.3. Experimental Procedure

**Figure 1:**
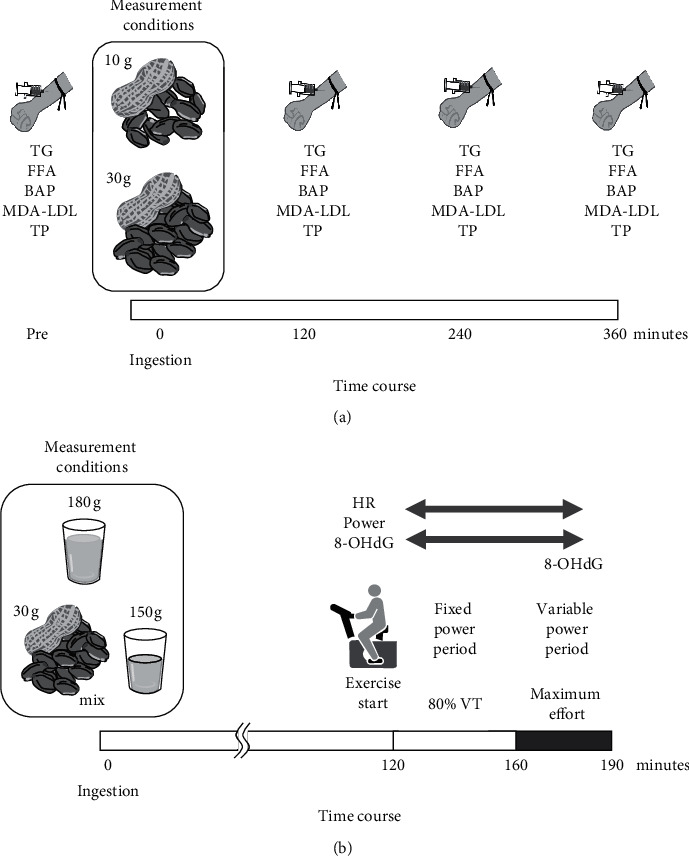
Schematics of (a) Experiment 1 and (b) Experiment 2. In Experiment 1, peanuts were consumed with the skin, and the experiment was conducted randomly in 10 g and 30 g conditions. In Experiment 2, the experiment was conducted randomly in the 30 peanuts and 150 g water smoothie and 180 g water conditions. TG: triglyceride, FFA: free fatty acid, BAP: biological antioxidant potential, MDA-LDL: malondialdehyde-modified LDL, and TP: serum total protein.

Blood samples were collected in the morning in a fasting state at 10 h after the last meal. We collected venous blood from the median cutaneous vein of the elbow. About 5 mL of venous blood was collected in a collection tube before and at 2, 4, and 6 h after ingesting 10 and 30 g of peanuts. A previous study has shown that the ingestion of 1 ounce (28.35 g) of peanuts has an influence on health [17]. Considering the difference in the body size between Western and Asian populations [18, 19], we decided to use 20 g as a standard. Accordingly, we set the ingestion conditions of 10 g and 30 g in Experiment 1. During the experiment, the participants were asked to read and/or watch a video while waiting for the blood collection point and not to exercise. Each of the participants was subjected to the ingestion condition once per week, with each participant being subjected to both conditions in a random order (Figure 1(a)). The tubes were allowed to stand for 1 h and were centrifuged at 3,000 rpm for 15 min. Furthermore, the serum was transferred to a separate tube and stored frozen. Subsequently, blood samples were sent to a commercial service company (LSI Medience Corp., Tokyo, Japan) for the measurement of triglyceride, FFA, MDA-LDL, and serum total protein levels. The biological antioxidant potential (BAP) test was performed in 10 *μ*L of serum sample using FREE® Carpe Diem (Wismerll, Tokyo, Japan). The BAP test assesses the reduction power of Fe^3+^ to Fe^2+^ and thus evaluates the ability to remove free radicals in a sample, where a higher value indicates a higher antioxidant capacity [20].

### 2.2. Experiment 2

#### 2.2.1. Participants

We included 12 healthy female participants (age: 20.5 ± 0.7 years, height: 160.1 ± 4.9 cm, weight: 54.2 ± 4.0 g, BMI: 21.2 ± 2.0 kg/m^2^), who provided informed consent for participation in the experiment. All participants were normotensive without neurological, cardiovascular, pulmonary, or kidney diseases; moreover, none of the participants had hip, knee, or ankle joint pain. No participant took any medication or supplements daily. Additionally, all of the participants in this study were students at a dietitian training school, and they were instructed to take almost the same food in both experiments. This study was approved by the Ethics Committee of the Yamagata Prefectural Yonezawa University of Nutrition Sciences (No. 30-5). The trial registration number was UMIN000044447.

#### 2.2.2. Experimental Procedure

*(1) Setup of the Load for the Endurance Exercise Test*. Each participant was subjected to a ventilatory threshold for determining the relative exercise load intensity used in the endurance exercise test. The exercise was performed using a bicycle ergometer (Aerobike 75XL III; Combi Co. Ltd., Tokyo, Japan) for the incremental workload test; furthermore, we measured the expiratory gas data during exercise using a breath-by-breath method with a respiratory metabolism monitoring system (Aeromonitor AE-310S, Minato Medical Science, Japan). The test was initiated at an initial power output of 50 W. Increments of 20 W were made every 2 min until exhaustion. In this test, participants were encouraged to maintain a cadence of 50 rpm, as prompted by a metronome. The criteria of volitional exhaustion were not able to maintain 45 rpm and respiratory exchange ratio values greater than 1.1 [21]. Subsequently, the ventilatory threshold of work (VT point) was determined from the oxygen uptake, carbon dioxide, and ventilation values [22]. After determining the VT point, to confirm the load of the endurance exercise test for each participant, one week before the endurance exercise test, we checked whether the participant could pedal for 40 min while maintaining a pedal speed of 60 rpm at an intensity of 80% of the VT.

*(2) Endurance Exercise Test*. The experimental conditions were as follows: ingesting a beverage containing 30 g of peanuts plus 150 g of water in a mixed (“peanut” condition) and ingesting 180 g of water (“control” condition), which was a single ingestion and crossover test (Figure 1(b)). The trial order was randomized. To avoid the fatigue effects, all measurements were performed at intervals of ≤5 days. On the test day, the individuals entered the laboratory 2 h before the exercise started, ingested a beverage, and remained in a resting state until the experiment started. The duration between peanut ingestion and exercise commencement was determined based on Experiment 1 as the time when the effects of peanut ingestion were observed. After 2 h, the endurance exercise test began with a bicycle ergometer (Cateye Ergociser EC-1600; CATEYE Co., Ltd., Japan). The pedaling exercise was performed for 70 min. The first 40 min involved fixed power, with the individuals pedaling at 80% intensity of VT for 40 min while maintaining a pedal speed of 60 rpm. The next 30 min involved variable power, with the individuals pedaling at their maximum effort [6]. The intra-exercise pedaling speed and load were recorded every minute, from which the workload was calculated. The intra-exercise heart rate was recorded every minute using a telemetry heart rate monitor. No water was provided during exercise. A 5-mL urine sample was collected before and after exercise within 30 min. Urine samples of 3 mL for 8-OHdG testing and 0.5 mL for creatinine testing were utilized for the analysis. The samples were frozen and sent to a commercial service company (LSI Medience Corp., Tokyo, Japan) for the measurement of 8-OHdG and creatinine levels. The measured 8-OHdG values were corrected by creatinine values.

### 2.3. Statistical Analysis

All values are presented as mean ± standard deviation (SD). In Experiment 1, to assess the effects of dose responses and blood biomarkers, we used 2 (conditions) × 4 (time points) repeated analysis of variance (ANOVA). If significant main effects or interactions were confirmed, we performed Tukey's post hoc test. In Experiment 2, between-group comparisons were performed using a paired *t*-test. The effect sizes were calculated using Gpower version 3.1.9.7. ANOVA, post hoc tests, *t*-tests, and 95% confidence interval (CI) were performed using commercially available statistical software (SPSS version 22.0J; IBM Japan Tokyo, Japan). Statistical significance was set at *p* < 0.05.

## 3. Results

### 3.1. Experiment 1

The blood triglyceride levels under the 10-g peanut-ingested condition were as follows: preingestion, 55.1 ± 21.0 mg/dL; 2 h after ingestion, 61.0 ± 16.8 mg/dL; 4 h after ingestion, 57.4 ± 21.7 mg/dL; and 6 h after ingestion. 47.5 ± 17.9 mg/dL. The blood triglyceride levels under the 30-g peanut-ingested condition were as follows: preingestion, 52.8 ± 13.8 mg/dL; 2 h after ingestion, 74.6 ± 23.3 mg/dL; 4 h after ingestion, 66.8 ± 30.2 mg/dL; and 6 h after ingestion, 50.4 ± 18.3 mg/dL.

**Figure 2:**
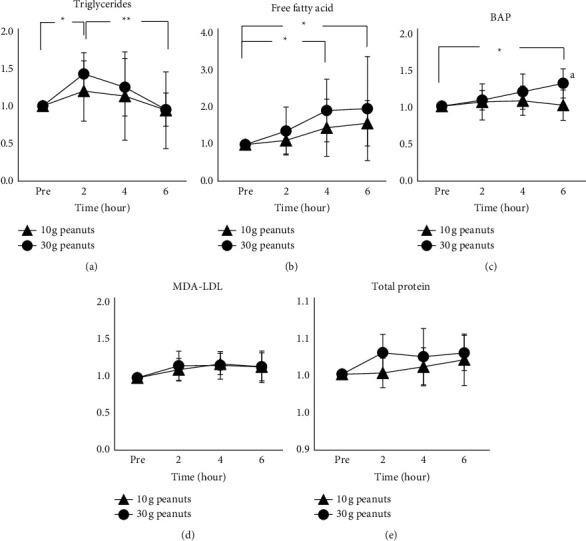
Relative changes in blood lipids. (a) Triglycerides, (b) free fatty acid, (c) BAP, (d) MDA-LDL, and (e) total protein. BAP: biological antioxidant potential; MDA-LDL: malondialdehyde-modified LDL. ^*∗*^Significant difference from preingestion (*p* < 0.05). ^*∗∗*^Significant difference from preingestion (*p* < 0.01). ^a^Significant difference (10 g peanuts vs. 30 g peanuts) (*p* < 0.01).

The results of 2-way ANOVA were [F(1,7) = 0.796, *p*=0.402, ES(hp2) = 0.102] for condition (10 g vs. 30 g), [F(3,21) = 4.193, *p* < 0.05, ES(hp2) = 0.375] for the time course, and [F(3,21) = 1.382, *p*=0.276, ES(hp2) = 0.165] for the condition × time course.  Figure 2(a) shows the relative postingestion changes in the values, indicating the time course change in each ingested condition. In the 30-g peanut-ingested condition, there were significant differences between the values obtained preingestion and 2 h after ingestion, and between the values obtained at 2 h and 6 h after ingestion [F (3,28) = 5.75, *p* < 0.01, ES (hp2) = 0.381; preingestion vs. 2 h; *p* < 0.05, 95% CI = 0.068–0.777, 2 h vs. 6 h; *p* < 0.01, 95% CI = 0.117–0.826].

The blood FFA levels under the 10-g peanut-ingested condition were as follows: preingestion, 0.59 ± 0.20 meq/L; 2 h after ingestion, 0.65 ± 0.24 meq/L; 4 h after ingestion 0.82 ± 0.35 meq/L; and 6 h after ingestion, 0.86 ± 0.27 meq/L. The blood FFA levels under the 30-g peanut-ingested condition were as follows: preingestion, 0.51 ± 0.17 meq/L; 2 h after ingestion, 0.67 ± 0.26 meq/L; 4 h after ingestion, 0.91 ± 0.32 meq/L; and 6 h after ingestion, 0.92 ± 0.32 meq/L.

The results of 2-way ANOVA were [F(1,7) = 1.919, *p*=0.208, ES(hp2) = 0.215] for condition (10 g vs. 30 g), [F(3,21) = 9.177, *p* < 0.01, ES(hp2) = 0.567] for the time course, and [F(3,21) = 1.076, *p*=0.381, ES(hp2) = 0.133] for the condition × time course.  Figure 2(b) shows the relative postingestion changes in the values, indicating the time course change in each ingested condition. In the 30-g peanut-ingested condition, there were significant differences among the values obtained preingestion, 4 h after ingestion, and 6 h after ingestion [F (3,28) = 4.57, *p* < 0.05, ES (hp2) = 0.329; preingestion vs. 4 h; *p* < 0.05, 95% CI = 0.079–1.734, preingestion vs. 6 h; *p* < 0.05, 95% CI = 0.129–1.784].

The blood BAP levels under the 10-g peanut-ingested condition were as follows: preingestion, 2,644.6 ± 387.8 *μ*mol/L; 2 h after ingestion, 2,731.1 ± 467.3 *μ*mol/L; 4 h after ingestion 2,781.4 ± 297.2 *μ*mol/L; and 6 h after ingestion, 2,627.6 ± 350.6 *μ*mol/L. The blood BAP levels under the 30-g peanut-ingested condition were as follows: preingestion, 2,548.4 ± 336.0 *μ*mol/L; 2 h after ingestion, 2,725.8 ± 196.5 *μ*mol/L; 4 h after ingestion, 2,990.2 ± 354.5 *μ*mol/L; and 6 h after ingestion, 3,292.4 ± 264.4 *μ*mol/L.

The results of 2-way ANOVA were [F(1,7) = 9.522, *p* < 0.05, ES(hp2) = 0.576] for condition (10 g vs. 30 g), [F(3,21) = 3.918, *p* < 0.05, ES(hp2) = 0.359] for the time course, and [F(3,21) = 9.240, *p* < 0.01, ES(hp2) = 0.569] for the condition × time course.  Figure 2(c) shows the relative postingestion changes in the values, indicating the time course change in each ingested condition. In the 30-g peanut-ingested condition, there were significant differences among the values obtained preingestion and 6 h after ingestion [F (3,28) = 3.15, *p* < 0.05, ES (hp2) = 0.253; preingestion vs. 6 h; *p* < 0.05, 95% CI = 0.022–0.518]. The 30-g peanut-ingested condition also showed a significant increase compared to the 10-g peanut-ingested condition at 6 hours after ingestion (*p* < 0.05, ES(d) = 1.096, 95% CI = 0.060–0.450).

The blood MDA-LDL levels under the 10-g peanut-ingested condition were as follows: preingestion, 111.7 ± 26.7 U/L; 2 h after ingestion, 121.2 ± 20.5 U/L; 4 h after ingestion 129.8 ± 25.6 U/L; and 6 h after ingestion, 124.5 ± 18.3 U/L. The blood FFA levels under the 30-g peanut-ingested condition were as follows: preingestion, 114.7 ± 36.5 U/L; 2 h after ingestion, 127.3 ± 24.0 U/L; 4 h after ingestion, 128.2 ± 24.0 U/L; and 6 h after ingestion, 125.0 ± 19.7 U/L.

The results of 2-way ANOVA were [F(1,7) = 0.119, *p*=0.741, ES(hp2) = 0.017] for condition (10 g vs. 30 g), [F(3,21) = 6.106, *p* < 0.01, ES(hp2) = 0.466] for the time course, and [F(3,21) = 1.864, *p*=0.167, ES(hp2) = 0.210] for the condition × time course.  Figure 2(d) shows the relative postingestion changes in the values, indicating the time course change in each ingested condition. There were no time course changes in either the 10-g or 30-g ingestion conditions.

The serum total protein levels under the 10-g peanut-ingested condition were as follows: preingestion, 7.45 ± 0.31 g/dL; 2 h after ingestion, 7.46 ± 0.35 g/dL; 4 h after ingestion 7.53 ± 0.38 g/dL; and 6 h after ingestion, 7.60 ± 0.50 g/dL. The blood FFA levels under the 30-g peanut-ingested condition were as follows: preingestion, 7.45 ± 0.39 g/dL; 2 h after ingestion, 7.66 ± 0.36 g/dL; 4 h after ingestion, 7.63 ± 0.47 g/dL; and 6 h after ingestion, 7.66 ± 0.42 g/dL.

The results of 2-way ANOVA were [F(1,7) = 1.112, *p*=0.327, ES(hp2) = 0.137] for condition (10 g vs. 30 g), [F(3,21) = 6.697, *p* < 0.01, ES(hp2) = 0.489] for the time course, and [F(3,21) = 0.984, *p*=0.419, ES(hp2) = 0.123] for the condition × time course.  Figure 2(e) shows the relative postingestion changes in the values, indicating the time course change in each ingested condition. There were no significant differences within the time course for each condition and between ingestion doses.

### 3.2. Experiment 2

#### 3.2.1. Maximum Incremental Load Test

Table 1 shows the exercise load at VT, exercise load and respiratory exchange ratio (RER) at all out, oxygen uptake at VT, peak oxygen uptake, and exercise duration evaluated from the maximal incremental load test.

#### 3.2.2. Endurance Exercise Test

**Figure 3:**
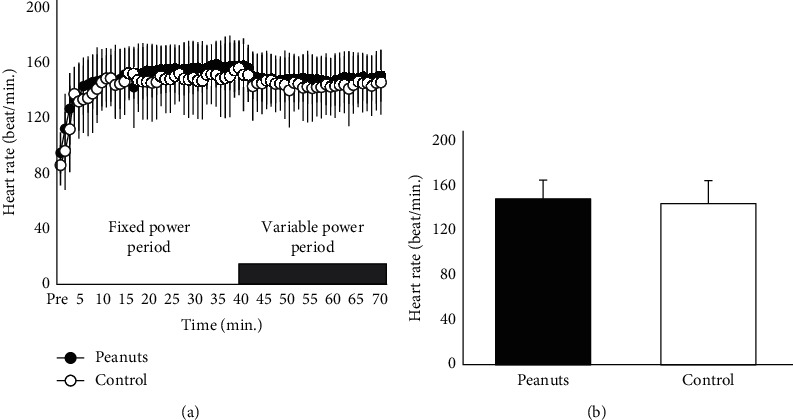
Changes in the heart rate. (a) Changes in the heart rate during pedaling exercise; (b) average heart rate during variable power periods.

*(1) Heart Rate*.  Figure 3(a) shows the change in the intra-exercise heart rate.  Figure 3(b) shows the average heart rate at the maximum effort interval (variable power). There was no significant difference in the fixed power between the peanut-ingested (149.0 ± 18.0 beat/min) and control (143.6 ± 20.0 beat/min) conditions (*p*=0.14, ES(d) = 0.409, 95% CI = −13.925–3.025). There was no significant difference in the variable power between the peanut-ingested (147.3 ± 16.6 beat/min) and control (143.1 ± 20.5 beat/min) conditions (*p*=0.18, ES(d) = 0.468, 95% CI = −9.670–1.766).

**Figure 4:**
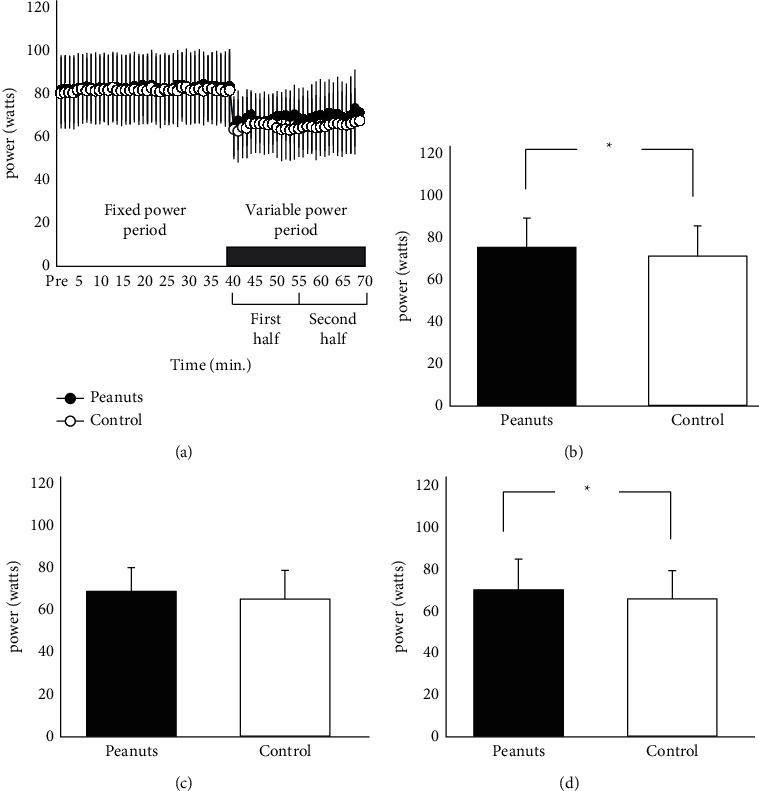
Changes in workload. (a) Changes in workload during pedaling exercise, (b) average workload during variable power periods, (c) average workload of the first half in the variable power period, (d) average workload of the second half in the variable power period. ^*∗*^Significant difference between peanut ingestion condition and control condition (*p* < 0.05).

*(2) Workload*. The changes in intra-exercise workload are shown in Figure 4(a). The average workload at the maximum effort interval (variable power) is shown in Figure 4(b). There was no significant difference in the fixed power between the peanut-ingested (82.5 ± 15.4 W) and control (81.4 ± 15.8 W) conditions (*p*=0.058, ES(d) = 0.620, 95% CI = −2.239–0.046). The variable power was significantly higher in the peanut-ingested condition (68.9 ± 12.7 W) than in the control condition (65.0 ± 13.3 W; *p*=0.048, ES(d) = 0.641, 95% CI = 0.032–7.773).  Figure 4(c) shows the average workload of the first half in the variable power period in the peanut-ingested (68.2 ± 11.2 W) and control (64.6 ± 13.7 W) conditions. There was no significant difference between the peanut-ingested and control conditions (*p*=0.121, ES(d) = 0.489, 95% CI = −1.109–8.234).  Figure 4(d) shows the average workload of the second half in the variable power period in the peanut-ingested (69.6 ± 14.7 W) and control (65.4 ± 13.4 W) conditions. The average workload of the peanut-ingested condition was significantly higher than that of the control condition (*p*=0.036, ES(d) = 0.682, 95% CI = 0.332–8.153).

**Figure 5:**
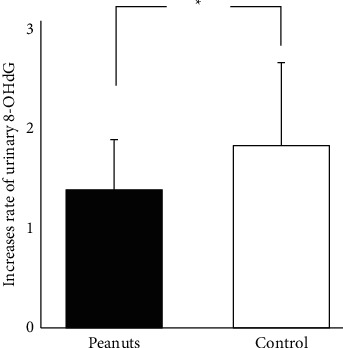
Comparison of the increase rate of urinary 8-OHdG. ^*∗*^Significant difference between peanut ingestion condition and control condition (*p* < 0.05).

*(3) Urinary 8-OHdG*. In the peanut-ingested condition, the pre- and postexercise urinary 8-OHdG values were 6.5 ± 2.2 ng/mgCr and 7.8 ± 1.4 ng/mgCr, respectively, while the corresponding values in the control condition were 4.5 ng/mgCr and 7.0 ± 2.3 ng/mgCr, respectively. In both conditions, there was a significant postexercise increase (peanut-ingested condition: *p*=0.048, ES(d) = 0.646, 95% CI = 0.010–2.590; control condition: *p* < 0.01, ES(d) = 1.119, 95% CI = 1.143–3.957).  Figure 5 shows the postexercise increase rate in each condition. The postexercise increase rate was significantly lower in the peanut-ingested condition (1.32 ± 0.48) than in the control condition (1.74 ± 0.79; *p*=0.048, ES(d) = 0.638, 95% CI = 0.002–0.842).

## 4. Discussion

This study showed that a single oral ingestion of high-oleic peanuts increased FFA and antioxidant levels; moreover, ingesting peanuts before prolonged exercise increased the workload during endurance exercise. This is the first study to demonstrate that ingesting high-oleic peanuts before exercise can effectively improve endurance performance. We speculated that FFAs and antioxidants may be involved in the observed enhancement of endurance performance.

This is the first study to show that a single oral ingestion of high-oleic peanuts significantly alters triglyceride, FFA, and antioxidant levels. In the 30-g peanut-ingested condition, triglyceride levels were significantly increased at 2 h after ingestion compared with those before ingestion. FFA levels were significantly increased at 4 h and 6 h after ingestion compared with before ingestion. Moreover, the antioxidant levels were significantly increased at 6 h after ingestion compared with before ingestion. In a study on the effects of a single oral ingestion of 85 g of peanuts with skin mixed into shakes in male individuals, Liu et al. reported a continuous increase in triglyceride levels after 2 h of ingestion compared with before ingestion [23]. The difference between the present and previous studies may be attributable to differences in the ingested volume. Regarding FFAs, since high-oleic peanuts contain a high amount of oleic acid [15] and the increment was dose-dependent, the FFA fluctuations could reflect the effects of high-oleic peanuts ingestion. Furthermore, the antioxidant effect may be influenced by the polyphenols and vitamin E in the high-oleic peanuts.

The exercise-related increased energy demand is mainly due to the supply of intermuscular glycogen, blood glucose, glycogenesis in the liver, and FFA from lipolysis. In low- and moderate-intensity exercise, energy supply systems are highly dependent on blood FFAs [24]. The exercise intensity in this study was considered moderate based on a proportion of the maximum heart rate [25]. Accordingly, the workload for the 30-minute maximal effort interval (variable power) was significantly higher than that under the control condition. Additionally, when the variable power interval was divided into the first and second halves of the period, a comparison of the workload between the control and high-oleic peanuts ingestion conditions showed no significant difference in the first half; however, a significant difference was observed in the second half period. Hagenfeldt and Wahren examined FFA oxidation using forearm exercises and found that 60 min of exercise increased the FFA uptake into skeletal muscle, with an increased uptake of linoleic and oleic acids relative to palmitic acid [26]. In our study, high-oleic peanuts contained high levels of oleic acid; moreover, ingesting this type of peanut increased FFA levels, which is an intra-exercise energy substrate, and contributed to improved exercise capacity.

High-oleic peanuts have high antioxidant activity [27], which may be involved in the observed improvement in endurance. Furthermore, there is an increase in reactive oxygen species (ROS) during endurance exercise [28, 29], which are associated with fatigue [30]. Antioxidants suppress ROS production [31, 32]. We observed that 8-OHdG, which is a biomarker of oxidative stress and DNA damage [33], was significantly decreased after peanut ingestion. It is known that 8-OHdG has high intraclass correlation coefficients, with good reproducibility and a good coefficient of variation, which makes it a suitable oxidative stress biomarker in spot urine samples [33, 34]. The observed improvement in endurance was attributed to the intra-exercise inhibition of ROS accumulation by the antioxidant effects of high-oleic peanut ingestion.

Finally, antistress effects may contribute to the observed improvement in endurance performance caused by ingesting high-oleic peanuts. Cortisol levels increase during prolonged exercise [35], which could result from exercise-related homeostasis disruption, including the metabolic utilization of substrates and normal vascular responses to the energy demands of active muscles during exercise. Coiro et al. reported that increased blood FFA levels during exercise suppressed exercise-induced cortisol secretion [36], which suggests that increased blood FFA levels may reduce the exercise-related homeostasis disruption. Although we did not measure stress hormone levels, the increase in FFA levels due to ingesting high-oleic peanuts may have reduced the exercise-related homeostasis disruption and improved endurance performance. Further studies on this point are warranted.

We observed that ingesting high-oleic peanuts before exercise improved endurance performance. The aforementioned factors could be involved in the mechanism of action underlying this phenomenon. However, as a major limitation of this study, the sample size was small. Therefore, future studies are needed to increase the sample size and to clarify whether these factors are involved, or whether one or more of them influence this phenomenon. Moreover, there is a need for future studies on the effects of chronic experiments.

## 5. Conclusions

Ingesting high-oleic peanuts before exercise increased blood FFA levels and improved endurance performance. This suggests that increased blood FFA levels due to the ingestion of high-oleic peanuts may serve as an energy substrate for lipid metabolism; moreover, the antioxidant effect of high-oleic peanuts may contribute to the improvement of endurance performance. More research is needed on the combination of other nutrients and the development of new intake strategies and their effects on endurance performance.

## Figures and Tables

**Table 1:** Results of the maximum incremental load test.

	Load at VT (W)	Load at all out (W)	RER at all out	Oxygen uptake at VT (ml/kg/min)	Peak oxygen uptake (ml/kg/min)	Exercise duration (min)
Mean	115.0	150.0	1.2	27.4	37.9	10.6
SD	19.3	17.1	0.07	4.8	6.6	2.3

VT, ventilatory threshold of work; RER, respiratory exchange ratio; SD, standard deviation.

## Data Availability

The data used to support the findings of this study are available from the corresponding author upon request.
